# Undergraduates short form video addiction and learning burnout association involving anxiety symptoms and coping styles moderation

**DOI:** 10.1038/s41598-025-09656-x

**Published:** 2025-07-07

**Authors:** Minghua Mao, Feng Liao

**Affiliations:** 1 School of Foreign Language, Suzhou University of Technology, Changshu, China; 2 School of Mathematics and Statistics, Suzhou University of Technology, Changshu, China

**Keywords:** Short-form video addiction, Learning burnout, Anxiety symptoms, Negative coping styles, Positive coping styles, Psychology, Human behaviour

## Abstract

The proliferation of short-form videos on social media has become a ubiquitous aspect of contemporary life. However, the pervasive use of these videos may potentially give rise to an addiction-like phenomenon. College students, in particular, have emerged as a significant demographic engaging with short-form videos, prompting a growing interest among scholars in understanding the potential implications of this phenomenon. This study aimed to examine the relationship and mechanism of action between short-form video addiction, anxiety symptoms, coping styles, and learning burnout among college students. The sample was obtained through convenience sampling, and the study utilized data from 523 college students. All variables were measured using empirical questionnaire instruments, and all the instruments had good reliability. Regression analysis was used to test the mediating effect of anxiety symptoms between short-form video addiction and learning burnout. PROCESS macro v3.5 for SPSS (Model 5) was adopted to test the moderated mediation. Results indicated that short-form video addiction was positively and significantly associated with both learning burnout and anxiety symptoms, and that anxiety symptoms mediated the relationship between short-form video addiction and learning burnout. Furthermore, negative coping styles significantly weakened the association between short-form video addiction and learning burnout. These results suggest that educational interventions should integrate digital literacy programs with mental health services to reduce the impact of addiction and provide targeted training in positive coping strategies to prevent learning burnout.

**Supplementary Information:**

The online version contains supplementary material available at 10.1038/s41598-025-09656-x.

## Introduction

Short-form video platforms (e.g., TikTok/Douyin), characterized by brief content typically lasting seconds to minutes^1^ now dominate digital engagement with 95.5% penetration among Chinese internet users^2^. Younger demographics are particularly affected—reports indicate that over 80% of Chinese university students actively use these platforms^3^. Their rapid adoption stems from two key algorithmic innovations: neural network-based recommendation systems that continuously optimize content matching^4^ and micro-length stimuli designed to trigger dopamine release through variable reward schedules^5,6^. Unlike traditional social media’s search-dependent interaction model, these platforms’ technological paradigm shift has led to emerging addiction patterns among users, particularly college students^6^. As these platforms achieve ubiquity, researchers have documented emerging addiction patterns^1,7–9^. Current evidence suggests addiction involves multiple psychological mechanisms, with two prominent but non-exclusive pathways: self-regulatory capacity (particularly self-efficacy) may buffer compulsive usage^10^ while anxiety-related vulnerabilities can amplify it^11^. Other factors like reward sensitivity^5^ and attentional control deficits^12^ likely interact with these core mechanisms.

These psychological processes intersect with platform affordances to drive resource depletion. Conservation of Resources (COR) theory^13^ frames this as a multidimensional loss phenomenon, where temporal resources are depleted through displacement of academic and productive activities^1,8^ cognitive resources become fragmented by rapid context-switching^12^ and emotional resources are drained through sustained exposure to algorithm-driven mood variability^5^. The immersive nature of short-form videos, facilitated by their technical design features, amplifies these effects, making them more addictive than typical social media platforms^14,15^. College students are especially vulnerable to this overuse^8,9^ which leads to mental health issues^16,17^ problematic behaviors^18^ and various forms of academic impairment, most notably academic burnout^19,20^.

Learning burnout may be one of the most significant negative effects of short-form video addiction on college students. Learning burnout refers to the emotional exhaustion, academic alienation, and a low sense of achievement caused by excessive learning demands^21^. Research has shown that when students are uninterested or lack motivation in their studies but feel compelled to engage in them, they may experience weariness, fatigue, depression, and frustration, leading to inappropriate avoidance behaviors related to learning^22^. A cross-sectional study of 22,983 Chinese university students revealed that over half suffered from academic burnout^23^. Learning burnout not only affects students’ academic performance and directly leads to a serious decline in academic quality, but also affects students’ future career development^24^. Multiple studies have demonstrated that higher levels of burnout are associated with lower academic achievement^25^ emotional dysregulation, sleep disorders^26^ depression^27^ and suicide^28^. It is therefore crucial to understand the factors that contribute to learning burnout to develop effective interventions. While prior research has identified problematic Internet use as a significant potential risk for learning burnout ^(14; 22; 24)^, most existing paradigms focus on screen time effects^29^. In contrast, short-form video platforms, characterized by temporal fragmentation and algorithmic reinforcement, may exacerbate cognitive depletion through rapid context-switching and attentional residue accumulation^12^. Given that short-form video addiction represents a specific type of Internet addiction^30^ it is plausible that it may exert a comparable influence on learning burnout. However, the relationship between college students’ short-form video addiction and learning burnout has received little empirical attention. This gap highlights the need for further research to explore how short-form video addiction specifically contributes to learning burnout among undergraduates.

### Short-form video addiction and learning burnout

The Conservation of Resources (COR) theory posits that when an individual is confronted with the prospect of resource loss, they will experience stress and doubt about their self-worth^13^. The excessive use of short-form videos by college students takes up a lot of study time, which leads to a loss of time and psychological resources due to the reduction of study time and lack of concentration in studying^1^. Due to the depletion of resources, students may experience feelings of concern and unease regarding their future development, a sense of inability to cope with their academic responsibilities^31^. Furthermore, when considered alongside the notion that learning burnout represents a state of physical and mental exhaustion, a loss of interest and motivation in learning due to the pressure and burden of academic pursuits, college students with short-form video addiction may also be susceptible to learning burnout^32,33^. Based on the Job Demands-Resources (JD-R) theory, the researchers considered mental resources to be limited for information processing. If the demands and mental resources are not compatible over time, it is easy to develop symptoms of burnout^34^. College students’ excessive investment of psychological resources in short-form videos results in insufficient resources being allocated to their learning demands, which in turn gives rise to learning burnout. Additionally, Liu et al.^35^ analyzed data obtained from 896 Chinese college students and found that short-form video addiction is positively correlated with stress, and stress is one of the important predictors of learning burnout^36^. Meta-analyses confirm there is a significant positive correlation between Internet overuse and burnout^32^ while short-form video addiction may be another subcategory of Internet addiction^1^. Based on the above, we speculate the following hypothesis.

H1: Short-form video addiction in college students is positively related to learning burnout.

### Anxiety symptoms as mediators

COR theory attributes video addiction-anxiety links to resource depletion^13^. Algorithm-driven short-form video platforms, characterized by instant reward feedback mechanisms and highly fragmented content presentation, continuously deplete users’ temporal and cognitive resources^1^. Studies have shown that when individuals allocate a substantial amount of psychological resources to short-form video use, their reserves for coping with academic stress are progressively depleted^13^. This state of resource imbalance engenders concerns and perceptions of uncertainty regarding future development^31^. Empirical evidence demonstrates a significant positive correlation between short-video addiction and anxiety levels^37^ with this association being partly realized through secondary resource depletion pathways, such as decreased sleep quality^38,39^.

The connection between anxiety symptoms and learning burnout can be elucidated in terms of the JD-R model. Anxiety symptoms increase students’ perceived cognitive load during learning^40,41^ while impairing their critical cognitive resources, such as attentional focus and information processing efficiency. This combination of increased demands and reduced resources can make students more susceptible to emotional exhaustion when tackling academic tasks^34^. Research has shown that students with higher anxiety levels require the mobilization of additional psychological resources to complete academic work^23^. Prolonged imbalance in resource allocation can ultimately result in the development of learning burnout.

Drawing on the integrated theoretical framework of Conservation of Resources (COR) theory and the Job Demands-Resources (JD-R) model, this study proposes a sequential mechanism whereby short-form video addiction initially depletes psychological resources, manifesting as increased anxiety symptoms^42,43^ which subsequently impairs students’ ability to maintain a balance between academic demands and available resources, ultimately culminating in learning burnout^40^. This cascading effect is further supported by empirical evidence demonstrating similar mediational pathways through psychological distress in related contexts of digital addiction^32^ adding to the plausibility of the proposed model. Therefore, we hypothesize:

H2: Anxiety symptoms may mediate the relationship between short-form video addiction and learning burnout.

### Coping styles as moderators

While anxiety symptoms may serve as a mediator linking short-video addiction to learning burnout, the magnitude of this linkage likely varies depending on moderating conditions that amplify or attenuate the observed relationships. Short-form video addiction tends to trigger a decline in students’ attention to learning and a backlog of learning tasks, thus increasing students’ learning pressure^20^. The Stress Coping Model (SCM) believes that the individual’s coping style has an important impact on the stress response. Coping style is characterized by conscious efforts to regulate cognition, behavior, physiology, emotion, and the environment in response to stress^44^. As an individual behavioral and cognitive strategy, coping styles can be divided into positive coping styles (including attempting to change and seeking support from others) and negative coping styles (including avoidance/withdrawal)^45,46^.

Short-form video addiction creates a dual challenge in academic settings by simultaneously depleting cognitive resources through attention fragmentation while increasing academic demands via task accumulation^20^. Within this context, coping styles play a pivotal moderating role in determining burnout outcomes, as evidenced by educational psychology research on academic stress and media studies on digital behavior patterns.

Positive coping styles may weaken the addiction-burnout relationship through several mechanisms. Educational research demonstrates that students employing active problem-solving strategies can better reorganize their study schedules to compensate for time lost to video use^26^ while those seeking social support may counteract the isolating effects of compulsive media consumption^47^. These strategies help restore the self-regulatory capacities that are often compromised by excessive short-form video use^13^ thereby buffering against burnout development.

Conversely, negative coping styles tend to strengthen the addiction-burnout link through distinct pathways. Media studies highlight how avoidance behaviors align with the dopamine-driven reward patterns of short-form platforms, creating a self-perpetuating cycle where academic tasks are increasingly postponed in favor of video consumption^48^. This pattern is exacerbated by the platforms’ always-accessible nature^20^. The resulting academic pressure then reinforces further media use as an escape mechanism^49^ accelerating the progression toward learning burnout.

Based on this information, it was hypothesized that:

H3: The positive coping styles may moderate the direct path between short-form video addiction and learning burnout.

H4: The negative coping styles may moderate the direct path between short-form video addiction and learning burnout.

### The present study

**Fig. 1:**
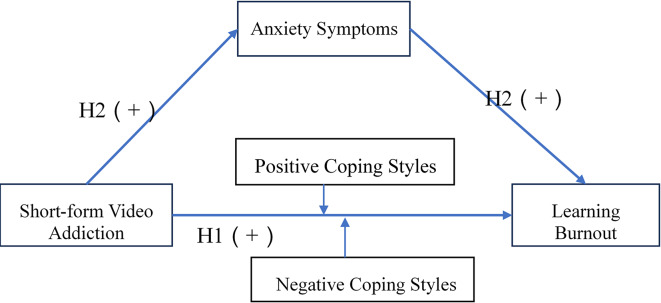
Theoretical model.

Building upon foundational work by Zhang et al.^1^ and Ye et al.^7,30^ that established initial frameworks for understanding short-form video usage patterns. Emerging scholarship has begun examining the relationships between short-form video addiction and various academic outcomes, including learning engagement^7,37^ academic well-being^9^ procrastination behaviors^20^ learning motivation^30^ and perceived stress^35^. While extensive research has established robust connections between generalized internet addiction and learning burnout^32^ the specific mechanisms linking short-form video addiction to learning burnout among college students remain underexplored. This gap persists despite the unique affordances of short-form video platforms, including their algorithmically driven reinforcement patterns and micro-reward structures^5^. This study examines the relationship between college students’ short-form video addiction and learning burnout, focusing on anxiety symptoms as mediators and coping styles as moderators. The theoretical model is shown in Fig. 1.

Expanding on the COR theory, compulsive short-form video consumption depletes temporal and psychological resources, triggering anxiety symptoms and subsequent learning burnout. The JD-R model further elucidates how these anxiety symptoms may exacerbate learning burnout by creating an imbalance between academic demands and depleted cognitive resources. The SCM delineates how coping styles moderate these detrimental effects. This integrates COR→ JD-R→SCM frameworks to explain how short-form video addiction contributes to learning burnout through a sequential resource-demand mechanism. This integrated approach offers a novel theoretical perspective for understanding the mechanisms underlying digital media addiction.

## Research method

### Participants and procedure

To delve into this topic more thoroughly, this study specifically focused on college students as participants. This decision was primarily because, unlike elementary and secondary school students who are under closer supervision, college students typically possess smartphones and enjoy greater autonomy in managing their time. Using the convenient sampling method, we randomly selected freshmen to seniors from a college in Jiangsu Province, eastern China, and distributed questionnaires through an online questionnaire platform. A total of 535 questionnaires were collected. Excluding invalid questionnaires with incomplete answers, short answer time, and single answers, 523 valid questionnaires were obtained with a validity rate of 97.76%. Among them, 323 (61.76%) were female students and 200 (38.24%) were male students. There were 254 freshmen, 109 sophomores, 115 juniors, and 45 seniors; and there were 345 students in science and engineering, 116 students in humanities and social sciences, and 62 students in other categories.

This study was approved by the Ethics Committee of Changshu Institute of Technology (Approval No KY0015). All methods were carried out in accordance with relevant guidelines and regulations. All participants were kept anonymous, and their information was securely stored. Informed consent was provided in the survey so that all participants were aware that they were participating in this study and that the data they provided would be presented anonymously.

### Questionnaire

#### Simple coping style scale

In this study, the simple coping style scale compiled by Xie^50^ was used to evaluate the individual’s coping style, including 20 items, which were divided into two dimensions: positive problem coping style and negative avoidance emotional coping style. The positive coping dimension is composed of 1–12 questions, and the negative coping dimension is composed of 13–20 questions. Four-level scoring was adopted. Example items are, “Ask for advice from family/friends/classmates” and “Take a break and set the problem aside temporarily.” After each coping style item, the columns were “not adopted, occasionally adopted, sometimes adopted, often adopted”, and the corresponding scores were 1, 2, 3, 4. The higher the score, the higher the tendency of positive coping style and negative coping style separately. The Cronbach’s alpha coefficient of the total scale in this study was 0.921. The Cronbach’s alpha coefficients of the two dimensions were 0.932 and 0.824, respectively, suggesting good reliability.

#### Short-form video addiction

In this study, we used the item adapted by Zhang et al. to measure short-form video addiction^1^. The scale includes 8 items. Example items are, “I have difficulties in focusing on my study or work due to this short-form video app” and “I have attempted to spend less time on this short-form video app but have not succeeded.” The value was measured for each item using a seven-point Likert scale that ranges from “1 = very disagree” to “7 = very agree”. The higher the total score, the higher the addiction tendency of the subjects to the short-form video. In this study, the Cronbach’s alpha coefficient for the scale was 0.923, with good reliability.

#### Learning burnout scale

In this study, the learning burnout scale for college students was used by Lian et al.^22^ which could better reflect the characteristics of burnout in the learning scene based on the situation of Chinese college students. The scale includes 20 items that form three dimensions of low mood, improper behavior, and low sense of accomplishment, which uses a 5-point Likert scale (1 = Completely inconsistent; 5 = Completely consistent). Example items are, “The knowledge I learn feels completely useless” and “I only study for exams or coursework requirements.” Topics 1,3,6,8,11,13,15,18 take reverse scoring. The higher the final total score, the higher the degree of learning burnout. The Cronbach’s alpha coefficient of the total scale in this study was 0.883, suggesting good reliability. The Cronbach’s alpha coefficients of the three dimensions were 0.881, 0.734, and 0.746, respectively.

#### Generalized anxiety symptoms scale

The 7-item Generalized Anxiety symptoms scale (GAD-7) developed by Löwe et al.^51^ was used to evaluate the anxiety symptoms level of college students. The scale includes a total of 7 items. Likert-4 points are scored (0= “not at all” to 3= “almost every day”). Example items are, “Can’t stop or control worrying” and “Get annoyed/irritable easily.” The total score for all items is calculated, with higher scores indicating a higher level of anxiety for the individual (in the last two weeks). In this study, the Cronbach’s alpha coefficient for the scale was 0.933, with good reliability.

### Data analysis

Sample size adequacy (*N* = 523) was evaluated through established psychometric and statistical standards. The sample exceeded Nunnally and Bernstein’s criterion of 10 cases per scale item for the 20-item burnout measure^52^. For regression analyses, it satisfied both Harris’s 20:1 participant-to-predictor ratio (accounting for 5 core variables) and Aiken and West’s recommendation of 75 additional cases per interaction term (accounting for 2 moderation effects), with remaining capacity for mediation tests^53,54^. This ensures that we have sufficient statistical power to detect meaningful relationships and effects within our data, thereby supporting the validity and reliability of our findings.

First, the exploratory factor analysis and the Harman single factor test were used, and then descriptive statistical analysis was performed, including the mean, standard deviation, and Pearson correlation coefficient of the core variables. Then, regression analysis was used to analyze the mediating effect of anxiety and the moderating effect of coping styles. In all analyses, we took short-form video addiction, learning burnout, anxiety symptoms, positive coping styles, and negative coping styles as variables, and all continuous variables were standardized.

## Results

### Test of exploratory factor analysis

The original questionnaire had a total of items, 20 for coping styles, eight for short-form video addiction, 20 for learning burnout, and seven for anxiety symptoms. SPSS 24.0 was used to conduct exploratory factor analysis (EFA). The results of the factor analysis indicated that after extracting the factors by Principal Axis Factoring and rotating the factors using Direct Oblimin, the factor loadings ranged from 0.351 to 0.745. Given that these loadings were above the traditional retention criteria (typically 0.30 or 0.40), all items were retained for subsequent analysis.

### Test of common method bias

Common method bias can be detrimental to the validity of the study. Harman’s Single-Factor Test is considered a practical method widely used in psychological empirical research^55^. Harman’s single-factor test showed the first common factor explained 20.605% (less than 40%) of the variance of all items, indicating that common method bias was not a problem.

### Descriptive statistics

Table 1 lists the mean, standard deviation, and Pearson correlation coefficient of each variable. It can be seen from Table 1 that short-form video addiction was positively correlated with generalized anxiety symptoms (*r* = 0.447, *p* < 0.01) and learning burnout (*r* = 0.280, *p* < 0.01), which indicated a higher degree of addiction to short-form video, the higher the degree of anxiety symptoms and learning burnout might be. Generalized anxiety symptoms were positively correlated with learning burnout (*r* = 0.201, *p* < 0.01). Positive coping style was negatively correlated with short-form video addiction (*r* = 0.220, *p* < 0.01), generalized anxiety symptoms (*r* = 0.197, *p* < 0.01), and learning burnout (*r*=-0.181), but not significantly. Negative coping style was positively correlated with short-form video addiction (*r* = 0.216, *p* < 0.01), generalized anxiety symptoms (*r* = 0.304, *p* < 0.01), and learning burnout (*r* = 0.159, *p* < 0.01).

**Table 1:** Descriptive statistics and correlation matrix of all variables.

	Mean	SD	SFVA	LB	AS	PCS	NCS
SFVA	3.027	1.303	1				
LB	2.944	0.374	0.280**	1			
AS	0.895	0.686	0.447**	0.201**	1		
PCS	2.804	0.583	-0.220**	-0.047	-0.197**	1	
NCS	2.325	0.560	0.216**	0.159**	0.304**	0.347**	1

*N* = 523. **p < 0.01. SFVA, short-form video addiction; LB, learning burnout; AS, anxiety symptoms; PCS, positive coping styles; NCS, negative coping styles.

### Testing of the mediating model

Regression analysis was used to test the mediating effect of anxiety symptoms between short-form video addiction and learning burnout (as shown in Table 2). The results showed that short-form video addiction is positively associated with both learning burnout (*β* = 0.280, *p* < 0.001, 95%CI= [0.057,0.104]) and anxiety symptoms (*β* = 0.447, *p* < 0.001, 95%CI= [0.195,0.276]). After anxiety symptoms are included in the equation, the positive effect of short-form video addiction on learning burnout is still significant. Short-form video addiction is positively related to learning burnout (*β* = 0.237, *p* < 0.001, 95%CI= [0.042,0.095]), while anxiety symptoms are still positively associated with learning burnout (*β* = 0.095, *p* = 0.043, 95%CI= [0.002,0.102]). The total effect of short-form video addiction on learning burnout was *β* = 0.28, with an indirect effect through anxiety symptoms of β = 0.043 (0.447 × 0.095). This mediation accounted for 15.4% (0.043/0.28) of the total effect.

**Table 2:** The mediating effect of generalized anxiety symptoms.

Variable	Equation 1(dependent variable: Learning burnout)				Equation 2(dependent variable: Anxiety symptoms)				Equation 3(dependent variable: Learning burnout)			
	β	t	*p*	95%CI	β	t	*p*	95%CI	β	t	*p*	95%CI
short-form video addiction	0.28	6.65	0.000	[0.057,0.104]	0.447	11.391	0.000	[0.195,0.276]	0.237	5.061	0.000	[0.042,0.095]
Anxiety symptoms	-				-				0.095	2.033	0.043	[0.002,0.102]
*R* ^2^	0.078				0.199				0.086			
*F*	44.226***				129.762***				24.312***			

***p < 0.001,*β* indicates the standardized regression coefficient.

**Fig. 2:**
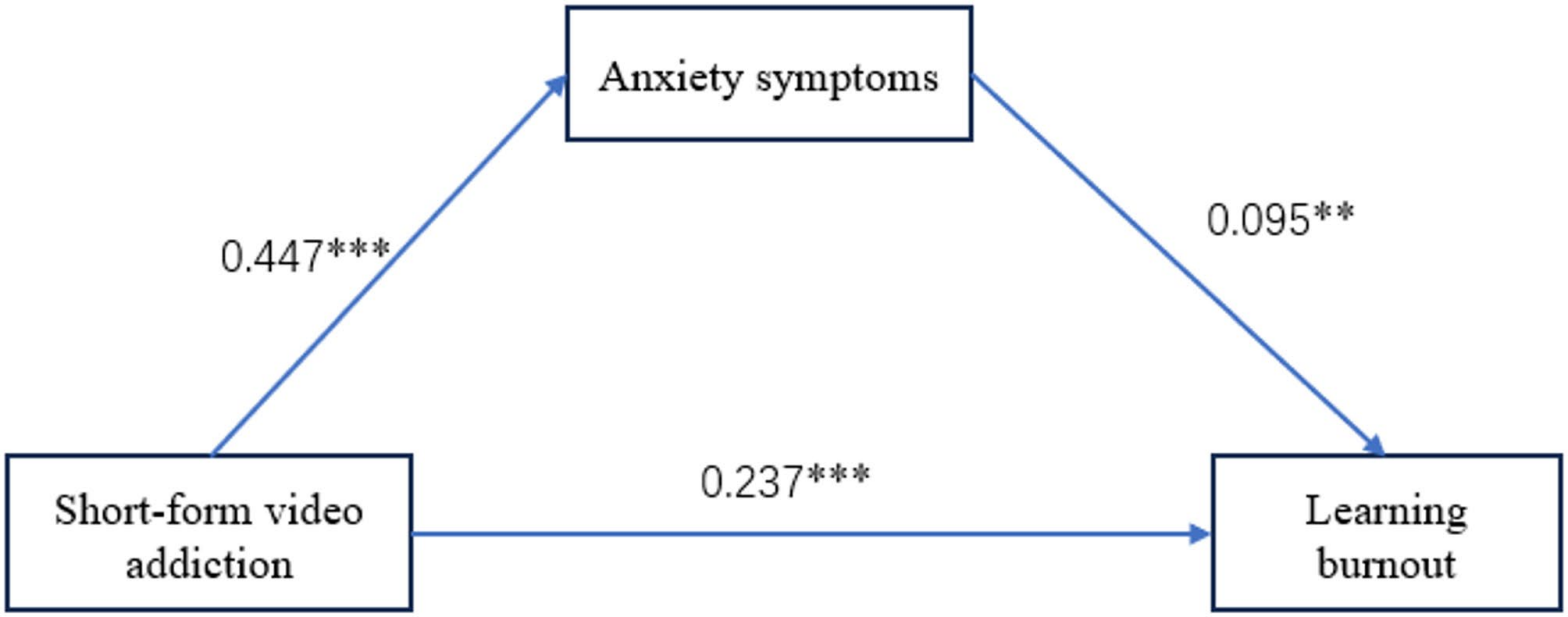
The path coefficients in the mediation model. **p < 0.01, ***p < 0.001.

To further clarify the mediating effect of anxiety symptoms in the relationship between short-form video addiction and learning burnout, bias-corrected Bootstrap was used to test the mediating effect. Repeat sampling 5000 times; if the 90% interval does not include the number 0, the mediating effect is significant. The results showed that the indirect effect of short-form video addiction on learning burnout reached a significant level (90% CI [ 0.004,0.083]). Results indicated that anxiety symptoms mediated the relationship between short-form video addiction and learning burnout. The mediation model is shown in Fig. 2.

### Testing of the moderated mediation

This study adopted PROCESS macro v3.5 by Andrew F. Hayes for SPSS (Model 5) to test the moderated mediation. The study variables were first standardized. The positive coping styles and negative coping styles were separately tested as moderators of the association between short-form video addiction and learning burnout. Table 3 shows the moderated mediating effect test results of negative coping style as a moderating variable. Short-form video addiction and the interaction between short-form video addiction and the negative coping styles significantly predicted learning burnout (*β* = 0.089, *p* < 0.001; *β*=-0.040, *p* = 0.005), which indicated that negative coping styles significantly weakened the effect of short-form video addiction on learning burnout. Hence, in the influence path of short-form video addiction on learning burnout, the moderating effect of negative coping styles on its direct path is significant, and hypothesis H4 was supported. However, the product of positive coping styles and short-form video addiction has no significant predictive effect on learning burnout, indicating that the moderating effect of positive coping style is not significant and hypothesis H3 was not supported.

**Table 3:** The moderated mediating effect of negative coping styles.

Outcome variable	Predictors	β	t	*p*	95%CI	*R* ^2^	F
Learning burnout	Short-form video addiction	0.089	5.041	0.000	[0.054,0.124]	0.106	15.367***
	Anxiety symptoms	0.030	1.652	0.099	[-0.006,0.066]		
	Negative coping styles	0.030	1.822	0.069	[-0.002,0.062]		
	Short-form video addiction x Negative coping styles	-0.040	-2.813	0.005	[-0.068,-0.012]		

***p < 0.001.

**Fig. 3:**
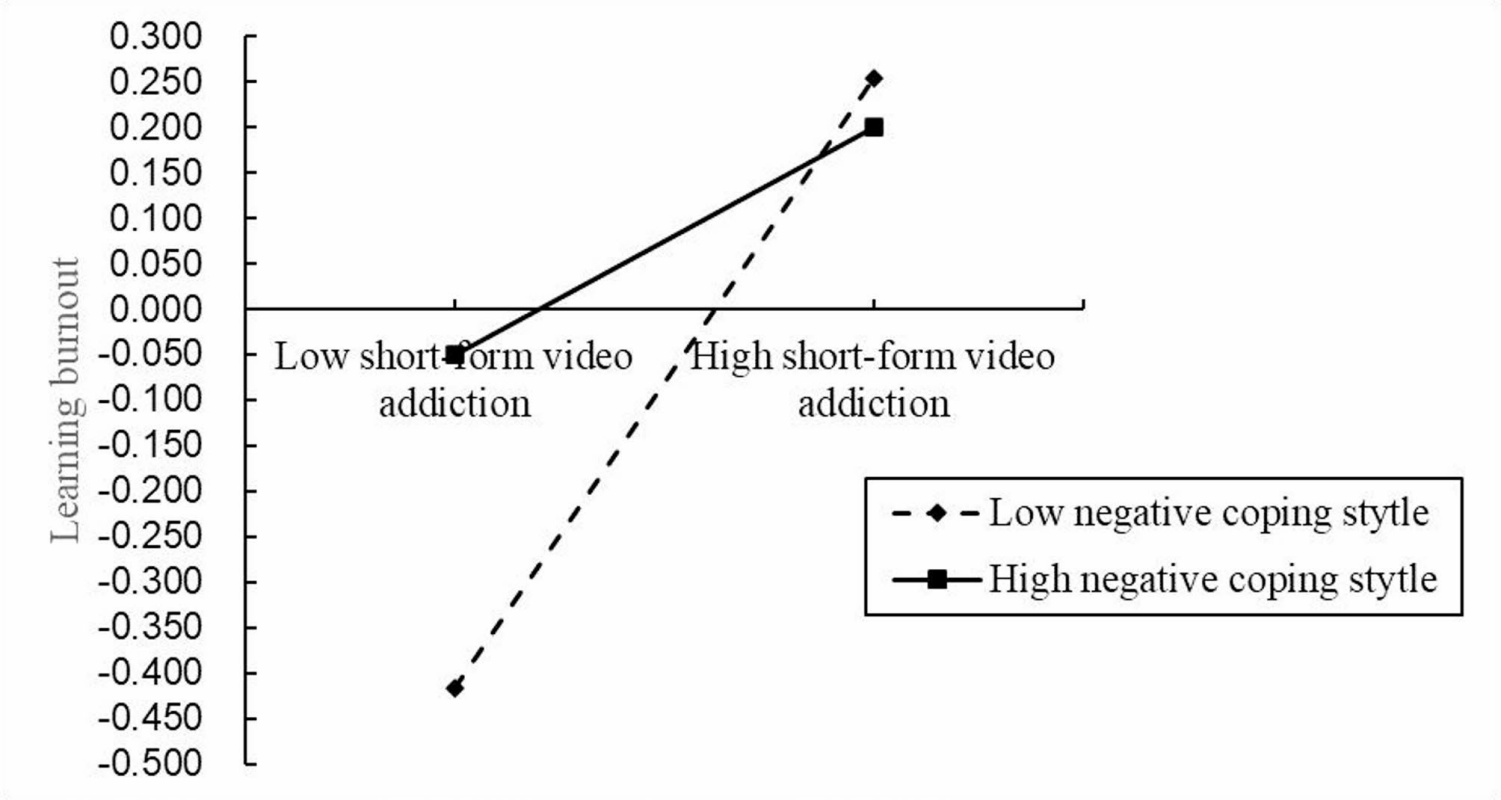
Moderating effect of negative coping style on short-form video addiction and learning burnout.

We plotted the results for learning burnout predicted by short-form video addiction for low (one standard deviation below the mean) and high (one standard deviation above the mean) negative coping styles (see Fig. 3). The moderating effects of different negative coping styles are shown in Table 4. The results showed that for college students with low negative coping, with the deepening of short-form video addiction, their learning burnout scores also increased significantly; for college students with high negative coping, with the deepening of short-form video addiction, their learning burnout scores also increased significantly, but the rate of increase slowed down significantly.

**Table 4:** The moderated effect of negative coping style.

Level of moderating variable	Regression coefficient	Standard error	t	*p*	95%CI
Low level	0.130	0.023	5.540	0.000	[0.084,0.176]
Mean	0.089	0.018	5.041	0.000	[0.054,0.124]
High level	0.049	0.022	2.193	0.029	[0.005,0.092]

## Discussion

This study constructs a moderated mediation model based on the Conservation of Resources (COR) theory, the Job Demands-Resources (JD-R) model, and the Stress Coping Model (SCM), and relevant empirical research findings. The present study examined the association between short-form video addiction and learning burnout, and the mediating and moderating effects on the association.

### The relationship between short-form video addiction and learning burnout

Results showed that short-form video addiction was positively associated with learning burnout, confirming hypothesis H1. Specifically, higher levels of short-form video addiction were linked to higher levels of learning burnout, consistent with prior research^32^. Zhang et al. proposed that excessive use of short-form video apps represents a state in which individuals spend substantial time on these platforms despite experiencing negative consequences^1^. College students with high levels of short-form video addiction tend to reduce their time dedicated to learning and gradually lose interest in activities beyond the consumption of short-form videos^33^ ultimately leading to learning burnout.

The findings align with and extend the COR theory. Short-form video addiction entails both temporal and psychological resource loss, as excessive use of these platforms decreases the time and cognitive energy available for academic tasks. This resource depletion is further worsened by the algorithmic reinforcement and rapid context-switching inherent in short-form video platforms, which foster a cycle of compulsive use and cognitive fragmentation. Consequently, students experience increased stress, which contributes to learning burnout.

### The mediating role of anxiety symptoms

The mediating role of anxiety symptoms in the relationship between short-form video addiction and learning burnout is a key finding of this study, supporting hypothesis H2. Short-form video addiction not only directly contributes to learning burnout but also indirectly exacerbates it by increasing levels of anxiety symptoms. This mediation mechanism aligns with the COR theory. In this context, excessive use of short-form video platforms depletes temporal and psychological resources, as students allocate significant time and cognitive energy to these platforms at the expense of academic tasks. This resource loss triggers anxiety symptoms, which, in turn, contribute to learning burnout.

The positive link between short-form video addiction and anxiety symptoms aligns with previous studies^37,43^. College students who are addicted to short-form videos often favor virtual engagement over academic responsibilities, leading to resource depletion and increased anxiety symptoms. Over time, this behavior cultivates social isolation and interpersonal challenges, which further intensify anxiety symptoms. The connection between anxiety symptoms and learning burnout is also well-established^56,57^. Anxiety symptoms hinder students’ ability to handle academic pressures, causing emotional exhaustion, decreased motivation, and a reduced sense of achievement, which are characteristics of learning burnout.

The findings shed further light on the underlying mechanisms between short-form video addiction and learning burnout, emphasizing the critical role of anxiety symptoms as a mediator. In the context of the new media era, short-form videos have become a popular medium for leisure and social interaction among college students^9^. However, their pervasive use poses significant risks to academic performance. The findings suggest that short-form video addiction directly undermines academic engagement and indirectly fosters learning burnout by heightening levels of anxiety symptoms.

### The moderating effect of coping styles

The results revealed that negative coping styles significantly moderated this relationship, while positive coping styles had no moderating effect. Negative coping styles significantly weakened the relationship between short-form video addiction and learning burnout. Specifically, individuals with higher levels of negative coping styles were less affected by the adverse impact of short-form video addiction on learning burnout, whereas those with lower levels of negative coping styles were more susceptible. This suggests that negative coping behaviors, such as avoidance or truancy, may mitigate the psychological and behavioral consequences of addiction, thereby reducing its predictive power for learning burnout.

From a theoretical perspective, this finding aligns with the Conservation of Resources (COR) theory, which posits that resource depletion leads to stress and maladaptive outcomes. Negative coping styles, while maladaptive, may serve as a temporary buffer against the resource depletion caused by short-form video addiction. By diverting attention away from academic stressors, negative coping styles may temporarily alleviate the emotional and cognitive burden associated with addiction, thereby weakening its impact on learning burnout. Empirical evidence supports this interpretation. College students who adopt negative coping styles are more likely to experience low mood, inappropriate conduct, and a lack of achievement^58,59^. However, in the context of short-form video addiction, these coping styles may paradoxically reduce the psychological strain imposed by academic demands, thereby diminishing the predictive power of addiction on learning burnout.

Contrary to hypothesis H3, positive coping styles did not moderate the relationship between short-form video addiction and learning burnout. This can be attributed to the psychological burden imposed by short-form video addiction, which depletes self-control resources and reduces individuals’ capacity to adopt positive coping strategies^60^. The constant suppression of the urge to engage with short-form videos consumes significant psychological energy, leaving learners with insufficient resources to implement effective coping mechanisms.

This study extends the COR theory by demonstrating how short-form video addiction depletes psychological and self-control resources, thereby limiting individuals’ ability to adopt positive coping strategies. Additionally, it highlights the role of negative coping styles as a buffer in the relationship between short-form video addiction and learning burnout, offering a nuanced understanding of how coping mechanisms influence academic outcomes in the digital age.

### Implications

The findings of this study have important theoretical and practical implications for understanding and addressing the relationship between short-form video addiction and learning burnout among college students. Theoretically, this study extends the Conservation of Resources theory by demonstrating how short-video addiction depletes time and psychological resources, leading to anxiety symptoms and learning burnout. The study also incorporates the Stress Coping Model, showing that negative coping styles act as a buffer, while positive coping styles do not moderate the effects of addiction. The mediating role of anxiety symptoms helps to explain how short-form video addiction leads to learning burnout, leading to a clearer understanding of the mechanisms involved.

The results of this study provide practical insights for addressing the relationship between short-form video addiction and learning burnout among college students. Educational institutions should implement interventions to reduce short-form video addiction, such as awareness campaigns and digital literacy programs, to help students manage their media consumption. Universities should prioritize mental health services, including counselling and stress management programs, to address anxiety and prevent it from escalating into learning burnout. Educators can promote adaptive coping strategies, such as problem-solving and seeking social support, through workshops or training sessions. In addition, students with low levels of negative coping styles may benefit from individual counselling or peer support groups to improve their coping skills and reduce the risk of learning burnout.

### Limitations

Several limitations should be acknowledged in this study. First, the use of self-report survey methods may be considered a limitation. As the variables are assessed through self-report questionnaires, the issue of methodological bias, which is prevalent in research, may emerge. Subsequent studies may employ a pairwise design to circumvent common method bias and augment the veracity of the extant findings. The duration and frequency of use of short-form video apps and addictive behaviors should be measured more objectively in future studies. Second, the use of cross-sectional data also prevents us from exploring the bidirectional effect between short-form video addiction and learning burnout. Learning burnout is a significant predictor of Internet addiction^32^ which can also be extended to short-form video addiction. Future studies should consider using empirical or longitudinal designs to validate the relationship between short-form video addiction and learning burnout. Third, the sample of the study may not be representative of the population of college students, as our sample was based on a convenience sampling method. In addition, the present study included only college students, and future research could expand the scope of the study to include, for example, adolescent students. Finally, the present study focused only on the mediating and moderating roles of two psychological resources in the relationship between short-form video addiction and learning burnout. Indeed, psychological resources such as resilience^24^ and self-efficacy^61^ may also be associated with the degree of short-form video addiction and learning burnout among college students, a relationship that can be further elucidated in subsequent studies. Resilience and self-efficacy will be regarded as new variables and their mechanisms in the influence of short-form video addiction on learning burnout will be explored. This will contribute to the existing literature on this topic.

## Conclusion

This study, based on the current status of short-form video usage among Chinese university students, reveals a dual-path mechanism through which short-form video addiction affects learning burnout, as demonstrated by an empirical analysis of 523 valid samples. The results show that: (1) short-form video addiction was not only directly and positively related to learning burnout; (2) but also exerts a significant indirect effect by exacerbating anxiety symptoms (indirect effect value 0.043, accounting for 15.4% of the total effect); (3) particularly under China’s high academic pressure context, negative coping styles exhibit a unique buffering effect. Based on these findings, we propose an intervention framework: at the preventive level, we recommend incorporating an “algorithm awareness” module into existing digital literacy curricula to help students recognize the addictive mechanisms in platform designs; at the intervention level, establishing peer support networks to lower psychological barriers to seeking professional help; and at the skill-building level, conducting scenario-based workshops to train specific coping styles.

## Electronic supplementary material

Below is the link to the electronic supplementary material.

Supplementary Material 1

## Data Availability

All data generated or analysed during this study are included in this published article and its supplementary information files.
